# Interaction effects of diabetes and brain-derived neurotrophic factor on suicidal ideation in patients with acute coronary syndrome

**DOI:** 10.1038/s41598-022-10557-6

**Published:** 2022-04-22

**Authors:** Wonsuk Choi, Ju-Wan Kim, Hee-Ju Kang, Hee Kyung Kim, Ho-Cheol Kang, Ju-Yeon Lee, Sung-Wan Kim, Young Joon Hong, Youngkeun Ahn, Myung Ho Jeong, Robert Stewart, Jae-Min Kim

**Affiliations:** 1 Department of Internal Medicine, Chonnam National University Hwasun Hospital, Chonnam National University Medical School, Hwasun, Republic of Korea; 2 Department of Psychiatry, Chonnam National University Medical School, 160 Baekseo-ro, Dong-gu, Gwangju, 61469 Republic of Korea; 3 Department of Cardiology, Chonnam National University Medical School, Gwangju, Republic of Korea; 4 Institute of Psychiatry, Psychology and Neuroscience, King’s College London, London, UK; 5 South London and Maudsley NHS Foundation Trust, London, UK

**Keywords:** Predictive markers, Acute coronary syndromes

## Abstract

Acute coronary syndrome (ACS) is related to an increased risk of suicide. Although both diabetes and the brain-derived neurotrophic factor (BDNF) pathway are closely associated with ACS and suicide, the effects of these factors on suicidal behavior in ACS patients have not been assessed. We investigated the individual and interaction effects of diabetes and BDNF-related markers, namely the serum BDNF (sBDNF) level and the *BDNF* Val66Met polymorphism, on suicidal ideation (SI) in ACS patients. The presence of diabetes was ascertained, and sBDNF levels and the presence of the *BDNF* Val66Met polymorphism were measured in 969 patients within 2 weeks after an ACS episode. 711 patients were followed up at 1 year after the ACS episode. SI was assessed using the relevant items of the Montgomery–Åsberg Depression Rating Scale at baseline (acute SI) and the 1-year follow-up (chronic SI). Significant individual effects of low sBDNF levels were found on acute SI. The presence of both diabetes and a low sBDNF level or the *BDNF* Met/Met genotype was associated with acute SI, with multivariate logistic regression analyses revealing significant interaction effects. The highest frequency of chronic SI was seen in diabetic patients with an sBDNF level in the lowest tertile or with the *BDNF* Met/Met genotype, although the interaction terms were not statistically significant. Our study suggests that the combination of diabetes and BDNF-related markers, such as the sBDNF level and the *BDNF* Val66Met polymorphism, might provide a useful predictor of acute SI in ACS patients.

## Introduction

Suicide is a global public health issue and is responsible for roughly 1 million deaths each year^1^. Acute coronary syndrome (ACS), which comprises unstable angina and myocardial infarction, is a serious life stressor that leads to a high risk of suicidal behavior^2–5^. Moreover, suicidal ideation (SI) after ACS is a risk factor for worse cardiac outcomes^6^. Given the association between ACS and suicide and the burdens of both, identifying risk factors for ACS-related suicidal behavior is important for developing effective prevention strategies for these patients.

Diabetes is a highly prevalent comorbidity among ACS patients and is associated with early mortality and major adverse cardiovascular events^7–9^. Diabetes also increases the risk of suicide^10,11^. However, whether diabetes is an additional risk factor for suicide in ACS patients has not been determined.

Brain-derived neurotrophic factor (BDNF) may contribute to suicide risk after ACS given its associations with both suicide and ACS. The involvement of BDNF in atherosclerosis and plaque instability^12^ was supported by a clinical study that reported reduced levels of BDNF in ACS patients^13^. BDNF has also been connected with suicidal behavior according to its role in maintaining synaptic and structural plasticity in the central nervous system^14,15^. However, inconsistent results regarding the association between the serum BDNF (sBDNF) level and suicidal behavior have been reported in previous studies. Some studies have reported that a low sBDNF level is associated with a higher suicide risk^16–18^, whereas others have not found such significant association^19,20^. Because the *BDNF* expression is controlled by a genetic polymorphism entailing substitution of valine by methionine at codon 66 (Val66Met) in the pro-BDNF molecule, which is related with reduced secretion of BDNF^21^, clinical studies have been carried out to evaluate the association between *BDNF* Val66Met polymorphism and suicide risk. However, discrepant results have been reported. Some studies have stated that the *BDNF* Met/Met genotype is associated with higher suicide risk^22–24^, whereas others have not found such significant association^25,26^.

Because diabetes and the BDNF pathway are closely related to ACS and suicide, respectively, and the level and function of BDNF are disrupted in diabetes^27,28^, suicidal behavior in ACS patients may be affected by a potentially complex relationship between diabetes and the BDNF pathway. However, this has not been studied to date. On the spectrum of suicidal behaviors, which range from SI and suicide attempts to completion^29^, SI has been used as a phenotype of suicidal behavior in ACS patients in previous studies^30,31^.

The goal of this study was to investigate the interaction effects of diabetes and BDNF-related markers, i.e., the sBDNF level and *BDNF* Val66Met polymorphism, on acute (within 2 weeks after an ACS episode) and chronic (1 year after an ACS episode) SI in a prospective cohort of Korean patients with ACS.

## Results

### Recruitment and treatment

Patient recruitment over the 1-year period is presented in Supplementary Fig. 1. Among the 1152 patients evaluated at baseline, 969 (84.1%) consented to offer blood samples. The baseline covariates did not differ significantly between those who agreed to offer blood samples and those who refused. Re-evaluation at 1 year after the ACS episode was performed in 711 (73.4%) of the 969 patients. Reasons for drop-out were loss to follow-up (N = 159), death (N = 9), refusal to participate (N = 32), and too unwell to participate (N = 13). Drop-out at the 1-year re-evaluation was significantly associated with older age and higher Killip class but not with diabetes, the sBDNF level, or presence of the *BDNF* Val66Met polymorphism.

### Baseline characteristics of presence of diabetes, sBDNF tertiles, presence of the *BDNF* Val66Met polymorphism, and acute SI

In the baseline sample (N = 969), diabetes was present in 191 (19.7%) participants. The median (interquartile range) and mean (standard deviation) sBDNF levels were 17.8 (7.0) and 17.6 (9.4) ng/mL, respectively. The sBDNF levels were divided into tertiles: high (20.50–52.61 ng/mL), middle (14.79–20.48 ng/mL), and low (1.37–14.79 ng/mL). Val/Val, Val/Met, and Met/Met genotypes were observed in 242 (25.0%), 498 (51.4%), and 229 participants (23.6%), respectively. Acute SI was present in 195 (20.1%) participants. These variables were compared according to diabetes presence in Table 1. Presence of diabetes was significantly associated with older age, female sex, lower education level, current unemployment, higher fasting glucose level, lower total cholesterol level, higher blood urea nitrogen (BUN) level, higher score on the Beck Depression Inventory (BDI), hypertension, lower rate of hypercholesterolemia, lower rate of current smoking, and lower left ventricular ejection fraction (LVEF). The characteristics were compared according to the sBDNF tertile and presence of the *BDNF* Val66Met polymorphism in Supplementary Tables 1 and 2, respectively. The sBDNF tertile was significantly associated with *BDNF* Val66Met polymorphism, age, current unemployment, hypertension, current smoking, and LVEF. Presence of *BDNF* Val66Met polymorphism was significantly associated with the sBDNF level, age, current unemployment, and BDI score. The characteristics were then compared according to presence of acute SI in Supplementary Table 3. The presence of acute SI was significantly associated with female sex, lower education level, rented housing, current unemployment, previous depression, and a higher score on the BDI. Eleven variables (age, sex, education, housing, currently unemployed, previous depression, BDI score, hypertension, hypercholesterolemia, current smoker, and LVEF) were selected for evaluation in the subsequent adjusted analyses.

**Table 1:** Comparisons of baseline characteristics according to diabetes presence in patients with acute coronary syndrome.

	Non-diabetes (N = 778)	Diabetes (N = 191)	Statistical coefficient^a^	P-value
Serum BDNF, mean (SD) ng/mL	18.0 (7.0)	17.1 (7.0)	t = 1.652	P = 0.099
BDNF Val66Met polymorphism, N (%) Met/Met	183 (23.5)	46 (24.1)	χ^2^ = 0.105	P = 0.949
**Socio-demographic characteristics**				
Age, mean (SD) years	57.1 (11.2)	62.4 (9.6)	t = -6.526	P < 0.001
Sex, N (%) female	199 (25.6)	70 (36.6)	χ^2^ = 9.372	P = 0.002
Education, mean (SD) years	10.0 (4.6)	9.2 (4.7)	t = 2.029	P = 0.043
Marital status, N (%) unmarried	113 (14.5)	28 (14.7)	χ^2^ = 0.002	P = 0.962
Living alone, N (%)	70 (9.0)	22 (11.5)	χ^2^ = 1.134	P = 0.287
Housing, N (%) rented	129 (16.6)	21 (11.0)	χ^2^ = 3.658	P = 0.056
Currently unemployed, N (%)	277 (35.6)	91 (47.6)	χ^2^ = 9.438	P = 0.002
**Laboratory assessment**				
Fasting glucose, mean (SD )mg/dL	125.9 (31.2)	174.7 (62.8)	t = -10.435	P < 0.001
Total cholesterol, mean (SD) mg/dL	187.1 (38.8)	178.6 (41.5)	t = 2.672	P = 0.008
BUN, mean (SD) mg/dL	14.8 (9.5)	17.3 (10.2)	t = − 3.106	P = 0.002
Creatinine, mean (SD) mg/dL	0.87 (0.27)	0.93 (0.35)	t = − 1.958	P = 0.051
**Depression characteristics**				
Previous depression, N (%)	25 (3.2)	9 (4.7)	χ^2^ = 1.107	P = 0.313
Family history of depression, N (%)	18 (2.3)	5 (2.6)	χ^2^ = 0.061	P = 0.805
BDI, mean (SD) score	9.6 (8.5)	11.5 (9.0)	t = − 2.746	P = 0.006
**Cardiac risk factors, N (%)**				
Previous ACS	31 (4.0)	8 (4.2)	χ^2^ = 0.017	P = 0.898
Family history of ACS	24 (3.1)	7 (3.7)	χ^2^ = 0.167	P = 0.683
Hypertension	338 (43.4)	120 (62.8)	χ^2^ = 23.114	P < 0.001
Hypercholesterolemia	403 (51.8)	83 (43.5)	χ^2^ = 4.271	P = 0.039
Obesity	336 (43.2)	79 (41.4)	χ^2^ = 0.209	P = 0.648
Current smoker	319 (41.0)	47 (24.6)	χ^2^ = 17.538	P < 0.001
**Current cardiac status**				
Killip class > 1, N (%)	129 (16.6)	39 (23.2)	χ^2^ = 1.576	P = 0.209
LVEF, mean (SD)	61.8 (10.8)	58.6 (12.7)	t = 3.237	P = 0.001
Troponin I, mean (SD) mg/dL	9.9 (15.1)	10.0 (14.2)	t = − 0.127	P = 0.899
CK-MB, mean (SD) mg/dL	17.6 (37.8)	16.4 (35.1)	t = 0.413	P = 0.680

^a^Independent two-sample t-test or χ^2^ test, as appropriate. BDNF, brain-derived neurotrophic factor; BUN, blood urea nitrogen; BDI, Beck Depression Inventory; ACS, acute coronary syndrome; LVEF, left ventricular ejection fraction; CK-MB, creatine kinase-MB. Diabetes was defined as a diagnosis of diabetes by a doctor or currently administering antidiabetic drugs.

### Effects of diabetes, sBDNF tertile, and *BDNF* Val66Met polymorphism on SI

The individual effects of diabetes, sBDNF tertile, and *BDNF* Val66Met polymorphism on SI are shown in Table 2. Diabetes was not significantly associated with SI. Compared with the high sBDNF tertile, the low tertile was significantly associated with acute SI in the adjusted analyses. Compared with the Val/Val genotype, the Val/Met and Met/Met genotypes were significantly associated with acute SI in the unadjusted analyses, but these associations were not significant after adjustment. In the 1-year follow-up evaluation (N = 711), presence of diabetes, sBDNF tertile, and presence of *BDNF* Val66Met polymorphism were not significantly associated with chronic SI in the unadjusted or adjusted analyses.

**Table 2:** Individual effects of diabetes, *BDNF* Val66Met polymorphism, and tertiles of serum BDNF levels on acute and chronic suicidal ideation in patients with acute coronary syndrome.

Exposure	Group	Acute suicidal ideation				Chronic suicidal ideation			
		N	No. (%) presence	OR (95% CI)		N	No. (%) presence	OR (95% CI)	
				Unadjusted	Adjusted^a^			Unadjusted	Adjusted^a^
Diabetes	Absent	778	148 (19.0)	1.00	1.00	569	63 (11.1)	1.00	1.00
	Present	191	47 (24.6)	1.39 (0.96–2.02)	1.11 (0.69–1.80)	142	24 (16.9)	1.63 (0.98–2.72)	1.50 (0.86–2.62)
sBDNF	High	323	60 (18.6)	1.00	1.00	228	26 (11.4)	1.00	1.00
	Middle	323	59 (18.3)	0.98 (0.66–1.46)	1.05 (0.65–1.70)	249	34 (13.7)	1.21 (0.71–2.08)	1.37 (0.77–2.42)
	Low	323	76 (23.5)	1.35 (0.92–1.97)	1.73 (1.08–2.79)*	234	27 (11.5)	0.99 (0.56–1.75)	1.04 (0.57–1.91)
*BDNF* genotype	Val/Val	242	36 (14.9)	1.00	1.00	173	18 (10.4)	1.00	1.00
	Val/Met	498	106 (21.3)	1.55 (1.02–2.34)*	1.36 (0.83–2.25)	378	47 (12.4)	1.22 (0.69–2.18)	1.07 (0.58–1.96)
	Met/Met	229	53 (23.1)	1.72 (1.08–2.75)*	1.40 (0.80–2.47)	160	22 (13.8)	1.37 (0.71–2.67)	1.25 (0.63–2.51)

^a^Adjusted for age, sex, education, housing, current unemployment, previous depression, BDI score, hypertension, hypercholesterolemia, current smoking, and LVEF. BDNF, brain-derived neurotrophic factor. *P < 0.05.

**Figure 1:**
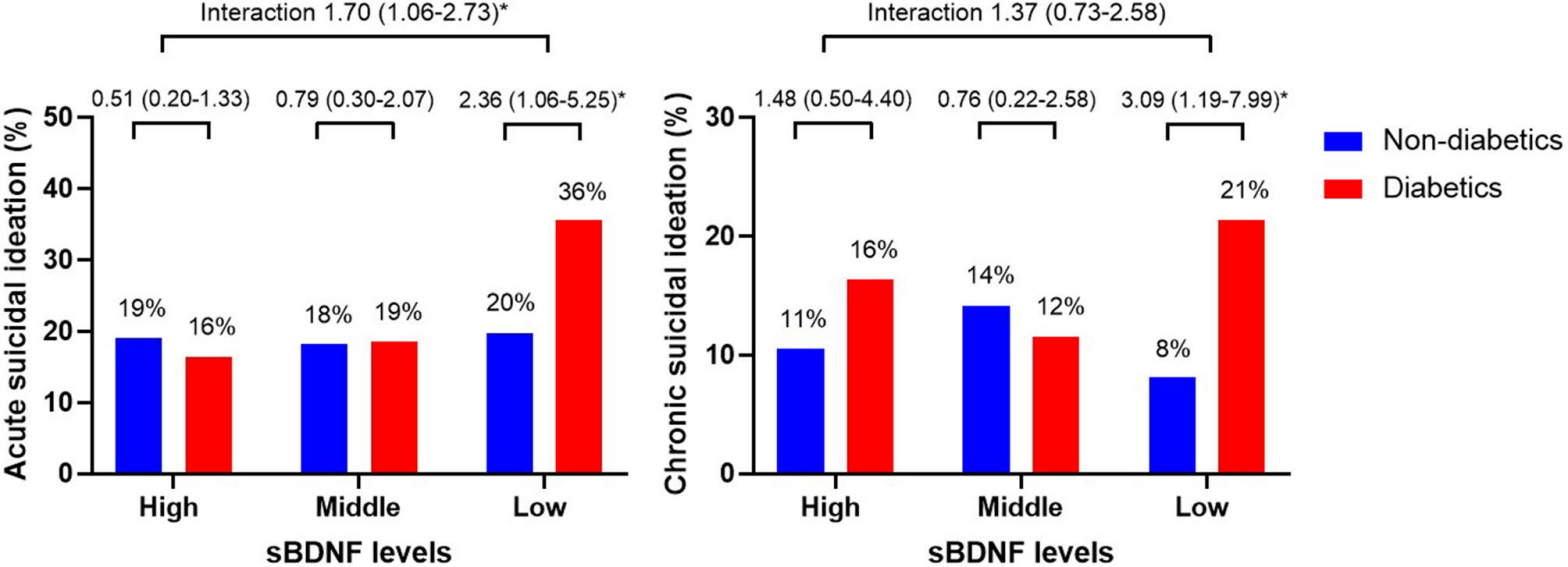
Interaction effects of diabetes and the sBDNF tertile on acute and chronic suicidal ideation in patients with acute coronary syndrome. Data are odds ratios (95% confidence interval) adjusted for age, sex, education, housing, current unemployment, previous depression, BDI score, hypertension, hypercholesterolemia, current smoking, and LVEF evaluated at baseline. *P < 0.05.

**Figure 2:**
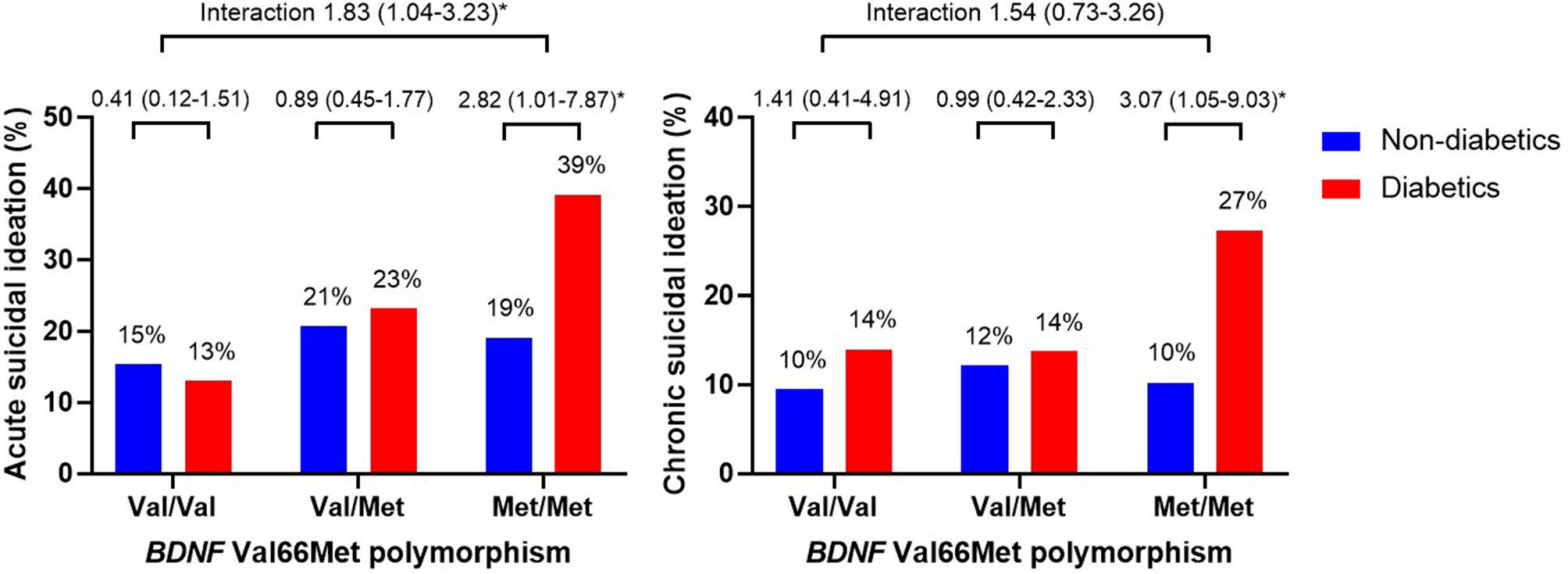
Interaction effects of diabetes and the *BDNF* Val66Met polymorphism on acute and chronic suicidal ideation in patients with acute coronary syndrome. Data are odds ratios (95% confidence intervals) adjusted for age, sex, education, housing, current unemployment, previous depression, BDI score, hypertension, hypercholesterolemia, current smoking, and LVEF evaluated at baseline. *P < 0.05.

The interaction effects of diabetes and sBDNF tertile on acute and chronic SI are shown in Fig. 1. Diabetes was significantly associated with both acute and chronic SI only among those in the low sBDNF tertile, and significant interactions with acute SI but not with chronic SI were found after adjustment for relevant covariates. The association between diabetes and acute SI in patients with the low sBDNF tertile was dependent on fasting glucose levels (Supplementary Fig. 2). The interaction effects of diabetes and *BDNF* Val66Met polymorphism on acute and chronic SI are shown in Fig. 2. Diabetes was significantly associated with both acute and chronic SI only in the presence of the *BDNF* Met/Met genotype, and a significant interaction with acute SI but not with chronic SI was found after adjustment for the relevant covariates. The association between diabetes and SI in patients with the *BDNF* Met/Met genotype was dependent on fasting glucose levels (Supplementary Fig. 3). The interaction effects of the sBDNF level and *BDNF* Val66Met polymorphism on acute and chronic SI are shown in Supplementary Fig. 2. No significant effects were found.

## Discussion

In this study, using data from a prospective study of Korean patients with ACS, we identified significant interaction effects of diabetes with both the sBDNF level and *BDNF* Val66Met polymorphism on acute SI, in that the incidence of acute SI was significantly higher in the presence of diabetes and a low sBDNF level or the *BDNF* Met/Met genotype compared with higher *BDNF* levels and other genotypes. These results were robust even after adjusting for the relevant covariates. Although the frequency of chronic SI was higher among diabetic patients with an sBDNF level in the lowest tertile or with the *BDNF* Met/Met genotype, the interactions were not statistically significant. With respect to individual effects, a significant association was found only between a lower sBDNF level and the presence of acute SI.

Diabetes reportedly increases the risk of suicide^10,11^, presumably due to diabetes-related complications and the high prevalence of mental disorders such as depression and anxiety^32–34^. These associations depend on the type of diabetes. Juvenile type 1 diabetes is closely related to all suicidal behaviors^35–37^, whereas in adult diabetics, the majority of whom have type 2 diabetes, inconsistent results have been reported regarding an increased risk of suicidal behavior. One study reported that diabetes was a risk factor for suicide completion^38^, whereas others reported no relationship^39–41^. The average and median age of diabetic patients included in this study were 62.4 years (standard deviation 9.6 years) and 64.0 years (interquartile range 14.0 years), respectively, and it is considered that most of them has type 2 diabetes. Our finding that diabetes exhibited no independent effect on acute or chronic SI was consistent with the results of many previous studies.

Because BDNF is involved in synaptic and structural plasticity in the brain^14,15^, abnormal BDNF function has been suggested as a biological pathway for suicide. Based on this suggestion, many clinical studies have evaluated the association between the BDNF pathway, including the sBDNF level and *BDNF* Val66Met polymorphism, and suicidal behavior, but the results have been inconsistent. Regarding the sBDNF level, some studies have reported an association between low sBDNF levels and a higher suicide risk^16–18^, whereas others have reported no such association^19,20^. Regarding the *BDNF* Val66Met polymorphism, some studies have found an association between the *BDNF* Met/Met genotype and a higher suicide risk^22–24^, whereas others have not^25,26^. The discrepant findings among previous studies may be the result of differences in the assessment methods for suicidal behavior and the presence and type of underlying mental disorders in the study subjects. However, discrepant results were also found between two studies with similar study designs that evaluated the association between *BDNF* Val66Met polymorphism and suicide attempts in depressed patients^24,25^. In the present study, high versus low tertiles of sBDNF levels showed an independent effect on acute SI, but the *BDNF* genotype did not, thus revealing discrepant results between the two BDNF-related markers. Considering our and others’ results comprehensively, BDNF-related markers alone seem to have limited value in predicting suicide risk.

In the present study, we identified an interaction effect of diabetes and a low sBDNF level or the *BDNF* Met/Met genotype on acute SI in ACS patients. In addition, although there were no significant interaction effects, the frequency of chronic SI was higher in diabetic patients with an sBDNF level in the lowest tertile or with the *BDNF* Met/Met genotype. A synergistic effect between diabetes and a low sBDNF level or the *BDNF* Met/Met genotype in our cohort is biologically plausible. A low peripheral BDNF level is associated with diabetes-related complications^42,43^. Because diabetes-related complications are related to a higher risk of SI^32^, diabetes may be predictive of SI only in subjects with low sBDNF levels or with the *BDNF* Met/Met genotype due to the high burden of those complications. In addition, because psychiatric diseases such as depression and anxiety are associated with increased suicidality in diabetics^32–34^, and a low sBDNF level and the *BDNF* Met/Met genotype are closely related to these psychiatric diseases^44^, diabetes may be a risk factor for SI only among subjects with a low sBDNF level or with the *BDNF* Met/Met genotype because of their high vulnerability to psychiatric diseases. Because ACS raises the risk of suicide^2–5^ and SI after ACS is linked to a worse illness outcome^6^, it’s critical to identify a high-risk group of ACS patients for suicide. However, there have been no research on biomarkers that can predict suicidal behavior in ACS patients to date. Our findings imply that assessing diabetes and BDNF-related markers in ACS patients could be used to screen high-risk groups for suicide.

It is noteworthy that interaction effects between diabetes and a low sBDNF level or the *BDNF* Met/Met genotype on SI were significant during the first 2 weeks but not at 1 year after an ACS episode. These results suggest that SI in ACS patients has several possible etiologies, which may vary depending on the time elapsed since the ACS diagnosis. Recently diagnosed ACS is associated with severe emotional and physical distress^45,46^, and the interaction effects of diabetes and altered BDNF-related markers may amplify these precipitating causes of acute SI. However, in the chronic phase, these interaction effects may disappear due to the greater influence of other factors on SI.

Several limitations to this study should be kept in mind in the interpretation of our results. First, SI, rather than suicide attempts or suicide completion, was evaluated as the primary outcome. Although SI is closely related to more severe suicidal behavior^47^, it is hard to generalize our findings to overall suicidal behavior in patients with ACS. However, since more severe types of suicidal behavior are rarely observed in this type of study, previous studies have also considered SI as a phenotype of suicidal behavior in ACS patients^30,31^. Second, SI was investigated using the “suicidal thoughts” item from the Montgomery–Åsberg Depression Rating Scale (MADRS), rather than using a formal instrument. However, the predictive validity of the suicide-related item for suicide attempts and suicide death has been demonstrated^48^, and the method used in this study has been applied before^49^. Third, diabetes assessment was based on self-report, which may have underestimated the actual prevalence of diabetes at baseline. Fourth, diabetes type and the burden of diabetes-related complications were not evaluated. However, most adults with diabetes in Korea are expected to have type 2 diabetes. Fifth, the follow-up rate for 1-year re-evaluation was relatively low compared with the baseline evaluation. Due to the poor prognostic characteristics of the ACS patients who were lost to follow-up, such as older age and a higher Killip class, this might have influenced the results. However, this likelihood is low because the baseline distributions of diabetes, sBDNF tertile, and *BDNF* Val66Met polymorphism did not differ by the 1-year re-evaluation follow-up status. Finally, recruitment was carried out at a single site, which may limit the study’s generalizability; however, this is also a strength as it guarantees consistency in patient evaluation and treatment.

This study has several strengths. It is the first prospective study to evaluate the interaction effects of diabetes and the BDNF pathway on SI. The BDNF pathway was evaluated using two markers, the sBDNF level and *BDNF* Val66Met polymorphism, which reinforced the validity of our results. All psychiatric and cardiovascular evaluations were carried out using well-validated scales. Moreover, acute SI and other covariates were assessed at similar time points (within 2 weeks of the ACS episode) in a large number of ACS patients, which reduced the risk of errors due to different examination times. The final strength was the successive recruitment of participants from all eligible ACS patients seen at the study hospital. This reduced the likelihood of selection bias and increased the generalizability of the outcomes in terms of screening patients at high risk of suicide.

Baseline diabetes and the high vs. low sBDNF level or *BDNF* Val/Val vs. Met/Met genotype showed multiplicative interactions in their associations with acute SI among patients with ACS. In addition, although the interaction effects were not significant, diabetes was associated with a higher frequency of chronic SI only in patients with a low sBDNF level or with the *BDNF* Met/Met genotype. These results suggest that the combination of diabetes and BDNF-related markers may increase the predictability of acute SI compared with each marker alone. Considering the preventive aspects, careful attention is needed for ACS patients who have both diabetes and altered BDNF-related markers; however, further studies are needed to evaluate whether additional suicide prevention practices may be beneficial in this subpopulation.

## Materials and methods

### Study overview and participants

All analyses were conducted using data from the Korean DEPression in ACS (K-DEPACS) study, which also included a nested randomized controlled trial, Escitalopram for DEPression in ACS (EsDEPACS) study (registration date: 08/01/2007, ClinicalTrial.gov registry number: NCT00419471). Design and results of the K-DEPACS study have been published^50–53^. The overview and participant recruitment algorithm for the present analysis are shown in Supplementary Fig. 1. Hospitalized ACS patients who satisfied the eligibility criteria (Supplementary Methods) at the Department of Cardiology of Chonnam National University Hospital, Gwangju, South Korea from 2006 to 2012 were consecutively recruited. The study cardiologists treated patients based on international guidelines for ACS management^54^. Patients who satisfied the inclusion criteria and consented to participate in the study were evaluated for baseline examinations as inpatients within 2 weeks (mean 6.3 standard deviation 2.4 days) after the ACS episode. Within this group, those who agreed to blood collections composed the baseline sample. They were followed up at 1-year after ACS for longitudinal evaluation. This study was approved by the Chonnam National University Hospital Institutional Review Board (CNUH I-2008-02-027). All participants reviewed the consent form and written informed consent was obtained.

### Exposure variables

#### Assessment of diabetes

Information with regard to history of diabetes was obtained at the baseline evaluation. Presence of diabetes was determined as a diagnosis of diabetes by a doctor or currently administering oral hypoglycemic agents or insulin treatment. Two abnormal test results from the same sample or two distinct test samples are required for diabetes diagnosis by fasting glucose level^55^. Since fasting glucose levels were measured once in an acute disease condition, which could induce hyperglycemia^56^, fasting glucose levels were inappropriate to use as a marker of diabetes diagnosis.

#### Serum BDNF level

Participants were instructed to fast (except water) overnight before blood sampling. They were then requested to sit quietly and relax for 25–45 min prior to obtaining the blood samples. The sBDNF level was quantified using the Quantikine^®^ ELISA Human BDNF Immunoassay (R&D Systems, Inc., Minneapolis, MN, USA) at the Global Clinical Central Lab (Yongin, Korea). The sBDNF level was classified into tertiles (high, middle, or low). Additionally, the patients were divided according to the median sBDNF level into higher and lower groups.

#### *BDNF* genotyping

The *BDNF* Val66Met polymorphism was determined as follows: DNA was extracted from venous blood using established protocols. The genotype was classified as Val/Val, Val/Met, or Met/Met. A detailed polymerase chain reaction protocol is presented in Supplementary Methods.

### Baseline covariates

Covariates associated with suicidal behavior in ACS patients in previous study^57^ were evaluated within 2 weeks after the ACS episode. An evaluation was conducted to collect information on age, sex, years of education, living status (living alone or not), type of residence (owned or rented), and current occupation (employed or not). Fasting glucose, total cholesterol, BUN, and creatinine levels were measured using the Hitachi Automatic Analyzer 7600 (Hitachi, Tokyo, Japan). To evaluate patients’ depression characteristics, personal and family histories of depression and BDI^58^ were ascertained. To evaluate patients’ cardiometabolic risk factors, the following characteristics were identified: personal and family histories of ACS, diagnosed hypertension, hypercholesterolemia according to fasting serum total cholesterol level (> 200 mg/dL) or a history of hyperlipidemia with ongoing treatment, obesity based on measured body mass index (BMI, > 25 kg/m^2^), and reported current smoking status. To evaluate current cardiac status, Killip classification^59^ was investigated and LVEF was estimated by echocardiography. Cardiac enzymes, including troponin I and creatine kinase (CK)-MB, were also measured.

### Outcome measure: suicidal ideation

SI was evaluated within 2 weeks after ACS (acute SI) and at 1-year after ACS (chronic SI) using the items of the MADRS for suicidal thoughts^60^. Participants were requested to rate whether they thought life was worth living or whether they had a suicide plan; scores ranged from 0 (life satisfaction) to 6 (explicit plans for suicide). Considering the previous study, the presence of SI was determined by a score of 2 (fleeting suicidal thoughts) or more^49^.

### Statistical analysis

The baseline data of diabetes presence, sBDNF tertiles, presence of the *BDNF* val66met polymorphism, and presence of acute SI were compared using independent *t*-tests or chi-square tests. Covariates were selected based on the variables representing statistical significance (P < 0.05) in the baseline characteristics analyses and potential collinearity between variables. The individual effects of diabetes (absent vs. present), the sBDNF level (high vs. middle or low), and the *BDNF* Val66Met polymorphism (Val/Val vs. Val/Met or Met/Met) on acute and chronic SI were analyzed using logistic regression before and after adjusting for covariates. The interaction effects between diabetes and the sBDNF level or *BDNF* Val66Met polymorphism and between the sBDNF level and *BDNF* Val66Met polymorphism on acute and chronic SI were analyzed by multinominal logistic regression after adjusting for potential covariates. All statistical tests were two-sided, and a P-value < 0.05 was considered to demonstrate statistical significance. Statistical analyses were conducted using IBM SPSS Statistics (version 25).

### Statement of ethics

All patients gave written informed consent to participate in the study and use their data. The study was conducted in accordance with the Helsinki Declaration of 1975, as revised in 2008 and approved by the Ethics Commission of the Chonnam National University Hospital Institutional Review Board (CNUH I-2008-02-027) as it uses de-identified data. EsDEPACS study was registered at ClinicalTrials.gov (registration date: 08/01/2007, ClinicalTrials.gov Identifier: NCT00419471).

## Supplementary Information

Supplementary Information.
