# Supplementary material for: Interaction effects of diabetes and brain-derived neurotrophic factor on suicidal ideation in patients with acute coronary syndrome
Source: Sci Rep. 2022 Apr 22;12:6602. doi: 10.1038/s41598-022-10557-6 (PMC9033782; doi:10.1038/s41598-022-10557-6)
Supplement: Supplementary file 1 — Supplementary Information. [file 41598_2022_10557_MOESM1_ESM.docx]

**Supplementary Information**

**Interaction effects of diabetes and brain-derived neurotrophic factor on suicidal ideation in patients with acute coronary syndrome**

Wonsuk Choi^a^, Ju-Wan Kim^b^, Hee-Ju Kang^b^, Hee Kyung Kim^a^, Ho-Cheol Kang^a^, Ju-Yeon Lee^b^, Sung-Wan Kim^b^, Young Joon Hong^c^, Youngkeun Ahn^c^, Myung Ho Jeong^c^, Robert Stewart^d,e^, and Jae-Min Kim^b^

^a^Department of Internal Medicine, Chonnam National University Hwasun Hospital, Chonnam National University Medical School, Hwasun, Korea, ^b^Department of Psychiatry, Chonnam National University Medical School, Gwangju, Korea, ^c^Department of Cardiology, Chonnam National University Medical School, Gwangju, Korea, ^d^ King’s College London, Institute of Psychiatry, Psychology and Neuroscience, London, UK, ^e^ South London and Maudsley NHS Foundation Trust, London, UK

**Supplementary Figure 1.** Participant recruitment and treatment


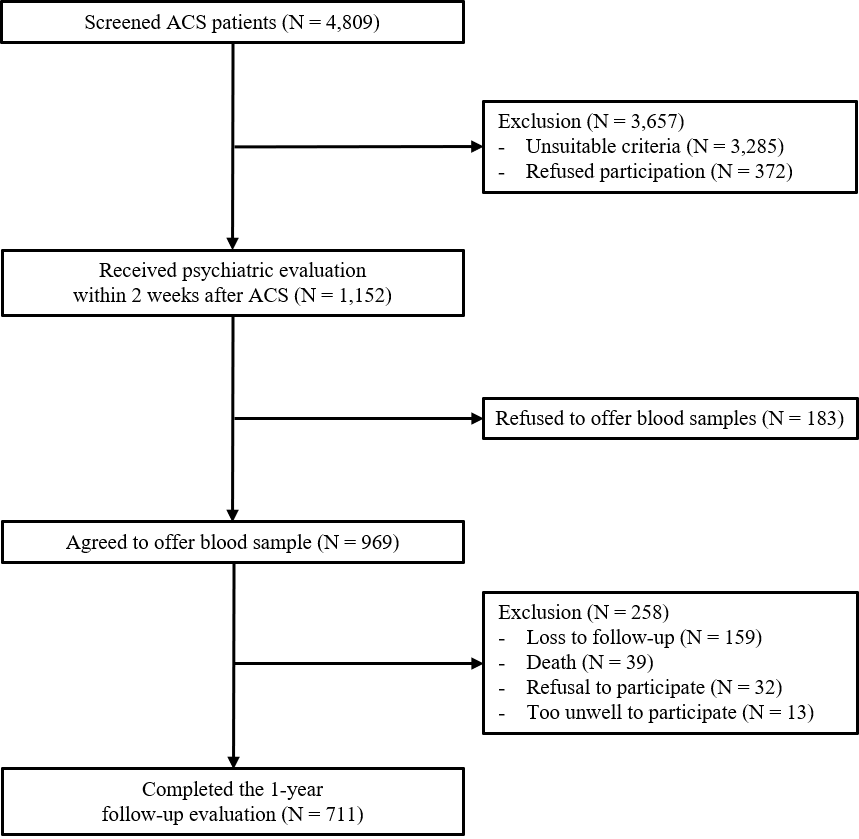


ACS, acute coronary syndrome.

**Supplementary Figure 2.**

Interaction effects of diabetes and the sBDNF tertile on acute and chronic suicidal ideation in patients with acute coronary syndrome.

Figure legends: Data are odds ratios (95% confidence interval) adjusted for age, sex, education, housing, current unemployment, fasting glucose, previous depression, BDI score, hypertension, hypercholesterolemia, current smoking, and LVEF evaluated at baseline. *P < 0.05.

**
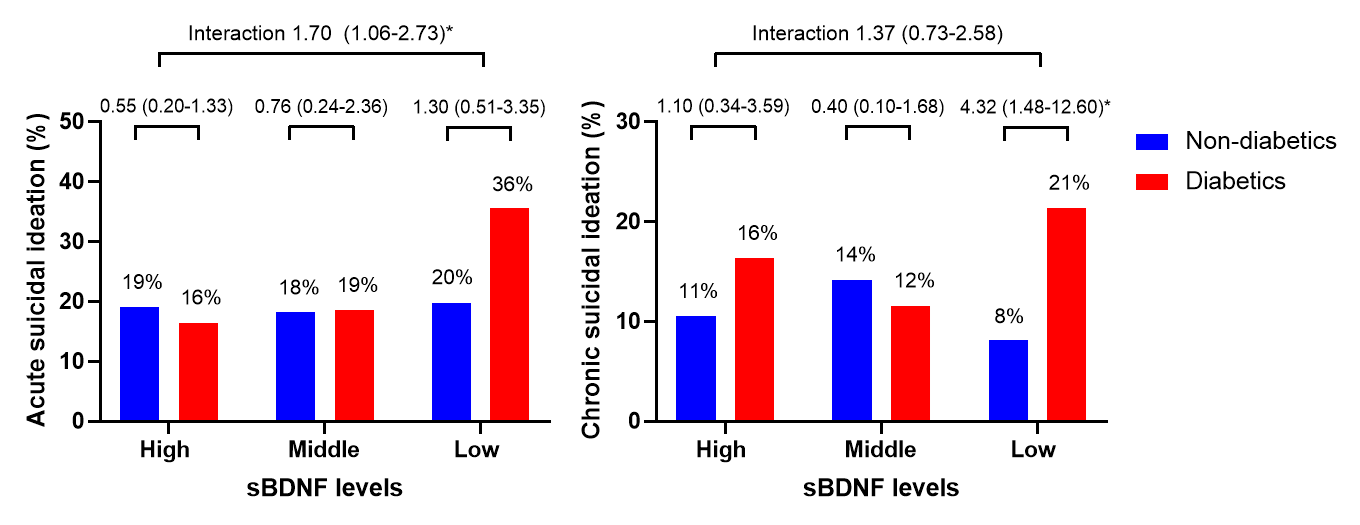
**

**Supplementary Figure 3.**

Interaction effects of diabetes and the *BDNF* Val66Met polymorphism on acute and chronic suicidal ideation in patients with acute coronary syndrome.

Figure legends: Data are odds ratios (95% confidence intervals) adjusted for age, sex, education, housing, current unemployment, fasting glucose, previous depression, BDI score, hypertension, hypercholesterolemia, current smoking, and LVEF evaluated at baseline. *P < 0.05.

**
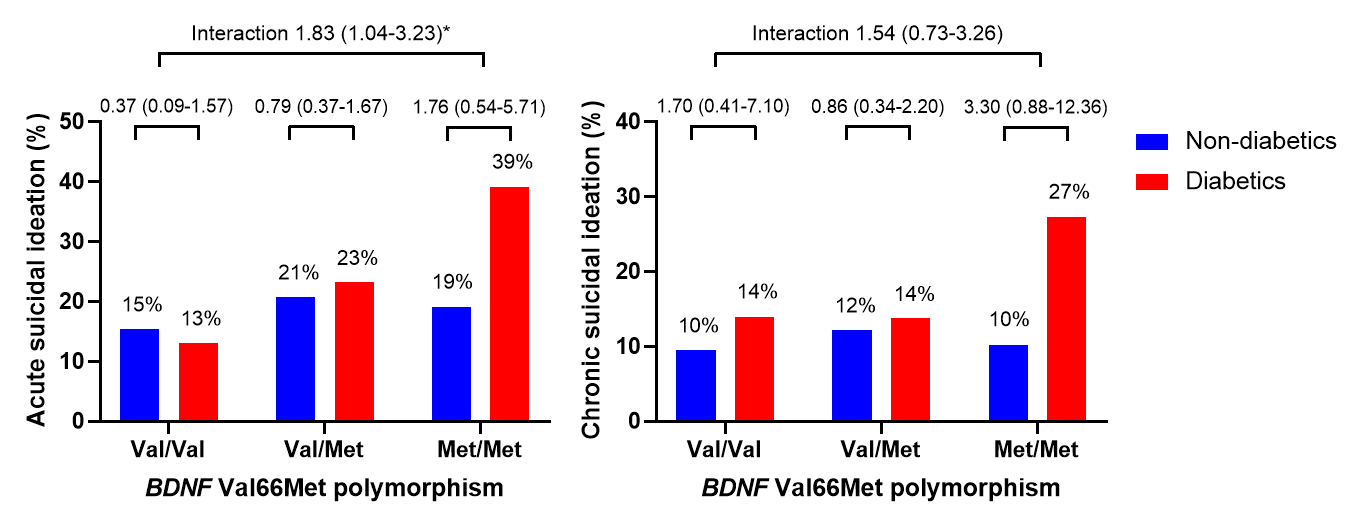
**

**Supplementary Figure 4.**

Interaction effects of the *BDNF* Val66Met polymorphism and sBDNF level on acute and chronic suicidal ideation in patients with acute coronary syndrome

Figure legends: Data are odds ratios (95% confidence intervals) adjusted for age, sex, education, housing, current unemployment, previous depression, BDI score, hypertension, hypercholesterolemia, current smoking, and LVEF evaluated at baseline.

*P < 0.05.


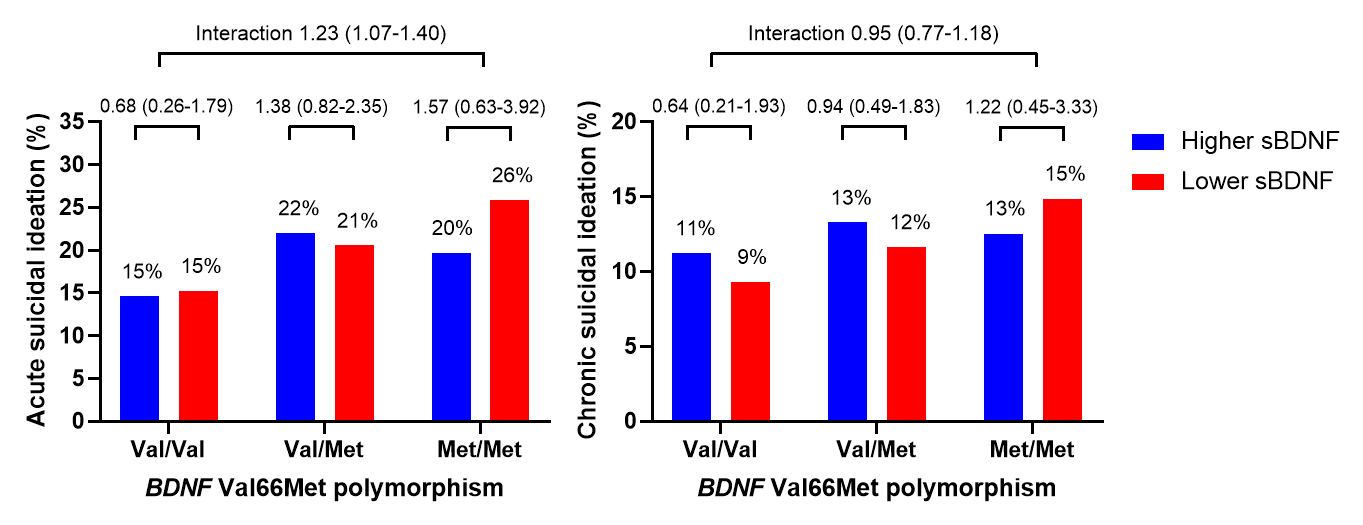


| **Supplementary Table 1.**  Comparisons of baseline characteristics according to the serum BDNF tertile in patients with acute coronary syndrome | | | | | | | |
| --- | --- | --- | --- | --- | --- | --- | --- |
|  |  | High (N = 323) | Middle (N = 323) | Low (N = 323) | | Statistical coefficient^a^ | P-value |
| Serum BDNF range (ng/mL) |  | 20.50-52.61 | 14.79-20.48 | 1.37-14.79 | |  |  |
| BDNF Val66Met polymorphism, N(%) Met/Met |  | 64 (19.8) | 74 (22.9) | 91 (28.2) | | χ^2^ = 28.907 | P < 0.001 |
| **Socio-demographic characteristics** |  |  |  |  | |  |  |
| Age, mean (SD) years |  | 57.2 (11.4)^b^ | 57.7 (10.8) | 59.7 (11.0) | | F = 4.600 | P = 0.010 |
| Sex, N (%) female |  | 90 (27.9) | 82 (25.4) | 97 (30.0) | | χ^2^ = 1.739 | P = 0.419 |
| Education, mean (SD) years |  | 9.9 (4.8) | 10.2 (4.6) | 9.5 (4.6) | | F = 1.895 | P = 0.151 |
| Marital status, N (%) unmarried |  | 38 (11.8) | 48 (14.9) | 55 (17.0) | | χ^2^ = 3.635 | P = 0.162 |
| Living alone, N (%) |  | 30 (9.3) | 28 (8.7) | 34 (10.5) | | χ^2^ = 0.673 | P = 0.714 |
| Housing, N (%) rented |  | 53 (16.4) | 46 (14.2) | 51 (15.8) | | χ^2^ = 0.615 | P = 0.735 |
| Currently unemployed, N (%) |  | 118 (36.5) | 110 (34.1) | 140 (43.3) | | χ^2^ = 6.344 | P = 0.042 |
| **Laboratory assessment** |  |  |  |  | |  |  |
| Fasting glucose, mean (SD )mg/dL |  | 134.7 (44.8) | 135.5 (45.1) | 136.3 (42.0) | | F = 0.102 | P = 0.903 |
| Total cholesterol, mean (SD) mg/dL |  | 185.9 (41.2) | 187.9 (37.3) | 182.3 (39.7) | | F = 1.642 | P = 0.194 |
| BUN, mean (SD) mg/dL |  | 15.1 (12.2) | 14.7 (5.5) | 16.0 (10.2) | | F = 1.463 | P = 0.232 |
| Creatinine, mean (SD) mg/dL |  | 0.87 (0.27) | 0.88 (0.29) | 0.91 (0.30) | | F = 1.879 | P = 0.153 |
| **Depression characteristics** |  |  |  |  | |  |  |
| Previous depression, N (%) |  | 11 (3.4) | 15 (4.6) | 8 (2.5) | | χ^2^ = 2.256 | P = 0.324 |
| Family history of depression, N (%) |  | 9 (2.8) | 10 (3.1) | 4 (1.2) | | χ^2^ = 2.761 | P = 0.251 |
| BDI, mean (SD) score |  | 10.2 (8.3) | 9.9 (8.5) | 9.8 (9.0) | | F = 0.187 | P = 0.830 |
| **Cardiac risk factors, N (%)** |  |  |  |  | |  |  |
| Previous ACS |  | 15 (4.6) | 12 (3.7) | 12 (3.7) | | χ^2^ = 0.481 | P = 0.786 |
| Family history of ACS |  | 15 (4.6) | 8 (2.5) | 8 (2.5) | | χ^2^ = 3.266 | P = 0.195 |
| Hypertension |  | 149 (46.1) | 134 (41.5) | 175 (54.2) | | χ^2^ = 10.960 | P = 0.005 |
| Diabetes |  | 61 (18.9) | 54 (16.7) | 76 (23.5) | | χ^2^ = 4.943 | P = 0.084 |
| Hypercholesterolemia |  | 165 (51.1) | 167 (51.7) | 154 (47.7) | | χ^2^ = 1.214 | P = 0.545 |
| Obesity |  | 137 (42.4) | 138 (42.7) | 140 (43.3) | | χ^2^ = 0.059 | P = 0.971 |
| Current smoker |  | 137 (42.4) | 125 (38.7) | 104 (32.2) | | χ^2^ = 7.350 | P = 0.025 |
| **Current cardiac status** |  |  |  |  | |  |  |
| Killip class >1, N (%) |  | 48 (14.9) | 58 (18.0) | 62 (19.2) | | χ^2^ = 2.247 | P = 0.325 |
| LVEF, mean (SD) |  | 62.5 (11.3) | 60.4 (11.0)^b^ | | 60.6 (11.5) | F = 3.462 | P = 0.032 |
| Troponin I, mean (SD) mg/dL |  | 9.1 (14.7) | 11.0 (16.1) | | 9.7 (13.9) | F = 1.380 | P = 0.252 |
| CK-MB, mean (SD) mg/dL |  | 14.8 (30.6) | 17.4 (35.1) | | 20.0 (44.7) | F = 1.585 | P = 0.206 |

^a^Analysis of variance (ANOVA) with post hoc Tukey’s test or χ^2^ test, as appropriate. ^b^P-value < 0.05 vs. high tertile, post hoc analysis. ^c^P-value < 0.05 vs. low tertile, post hoc analysis. BDNF, brain-derived neurotrophic factor ; BUN, blood urea nitrogen; BDI, Beck Depression Inventory; ACS, acute coronary syndrome; LVEF, left ventricular ejection fraction; CK-MB, creatine kinase-MB

| **Supplementary Table 2.**  Comparisons of baseline characteristics according to presence of the *BDNF* Val66Met polymorphism in patients with acute coronary syndrome | | | | | | | |
| --- | --- | --- | --- | --- | --- | --- | --- |
|  |  | Val/Val (N = 242) | Val/Met (N = 498) | Met/Met (N = 229) | | Statistical coefficient^a^ | P-value |
| Serum BDNF, mean (SD) ng/mL |  | 20.0 (6.8) | 17.3 (6.7)^b^ | 16.8 (7.2)^b^ | | F = 15.721 | P < 0.001 |
| **Socio-demographic characteristics** |  |  |  |  | |  |  |
| Age, mean (SD) years |  | 59.2 (10.9) | 57.2 (11.1) | 59.3 (11.2) | | F = 3.879 | P = 0.021 |
| Sex, N (%) female |  | 66 (27.3) | 131 (26.3) | 72 (31.4) | | χ^2^ = 2.102 | P = 0.350 |
| Education, mean (SD) years |  | 9.7 (4.6) | 10.1 (4.6) | 9.4 (4.8) | | F = 1.780 | P = 0.169 |
| Marital status, N (%) unmarried |  | 32 (13.2) | 80 (16.1) | 29 (12.7) | | χ^2^ = 1.916 | P = 0.384 |
| Living alone, N (%) |  | 22 (9.1) | 53 (10.6) | 17 (7.4) | | χ^2^ = 1.953 | P = 0.377 |
| Housing, N (%) rented |  | 34 (14.0) | 86 (17.3) | 30 (13.1) | | χ^2^ = 2.588 | P = 0.274 |
| Currently unemployed, N (%) |  | 106 (43.8) | 172 (34.5) | 90 (39.3) | | χ^2^ = 6.156 | P = 0.046 |
| **Laboratory assessment** |  |  |  |  | |  |  |
| Fasting glucose, mean (SD )mg/dL |  | 132.1 (39.4) | 135.9 (42.9) | 138.0 (50.4) | | F = 1.116 | P = 0.328 |
| Total cholesterol, mean (SD) mg/dL |  | 185.6 (42.1) | 183.7 (37.6) | 188.7 (40.6) | | F = 1.270 | P = 0.281 |
| BUN, mean (SD) mg/dL |  | 14.5 (5.3) | 15.3 (10.6) | 16.0 (11.1) | | F = 1.371 | P = 0.254 |
| Creatinine, mean (SD) mg/dL |  | 0.86 (0.26) | 0.89 (0.29) | 0.90 (0.32) | | F = 1.349 | P = 0.260 |
| **Depression characteristics** |  |  |  |  | |  |  |
| Previous depression, N (%) |  | 5 (2.1) | 22 (4.4) | 7 (3.1) | | χ^2^ = 2.841 | P = 0.242 |
| Family history of depression, N (%) |  | 4 (1.7) | 15 (3.0) | 4 (1.7) | | χ^2^ = 1.807 | P = 0.405 |
| BDI, mean (SD) score |  | 8.9 (7.8) | 10.2 (8.9) | 10.8 (8.7)^b^ | | F = 3.350 | P = 0.035 |
| **Cardiac risk factors, N (%)** |  |  |  |  | |  |  |
| Previous ACS |  | 7 (2.9) | 27 (5.4) | 5 (2.2) | | χ^2^ = 5.329 | P = 0.070 |
| Family history of ACS |  | 9 (3.7) | 16 (3.2) | 6 (2.6) | | χ^2^ = 0.459 | P = 0.795 |
| Hypertension |  | 110 (46.5) | 238 (47.8) | 110 (48.0) | | χ^2^ = 0.428 | P = 0.807 |
| Diabetes |  | 46 (19.0) | 99 (19.9) | 46 (20.1) | | χ^2^ = 0.105 | P = 0.949 |
| Hypercholesterolemia |  | 114 (47.1) | 244 (49.0) | 128 (55.9) | | χ^2^ = 4.185 | P = 0.123 |
| Obesity |  | 88 (36.4) | 224 (45.0) | 103 (45.0) | | χ^2^ = 5.504 | P = 0.064 |
| Current smoker |  | 93 (38.4) | 197 (39.6) | 76 (33.2) | | χ^2^ = 2.768 | P = 0.251 |
| **Current cardiac status** |  |  |  |  | |  |  |
| Killip class >1, N (%) |  | 35 (14.5) | 94 (18.9) | 39 (17.0) | | χ^2^ = 2.232 | P = 0.328 |
| LVEF, mean (SD) |  | 61.8 (11.5) | 60.7 (11.6) | | 61.7 (10.2) | F = 1.154 | P = 0.316 |
| Troponin I, mean (SD) mg/dL |  | 9.1 (13.1) | 10.6 (15.8) | | 9.1 (14.6) | F = 1.273 | P = 0.280 |
| CK-MB, mean (SD) mg/dL |  | 15.0 (32.0) | 18.1 (39.2) | | 18.3 (38.1) | F = 0.672 | P = 0.511 |

^a^Analysis of variance (ANOVA) with post hoc Tukey’s test or χ^2^ test, as appropriate. ^b^P-value < 0.05 vs. Val/Val in the post hoc analysis. BDNF, brain-derived neurotrophic factor; BUN, blood urea nitrogen; BDI, Beck Depression Inventory; ACS, acute coronary syndrome; LVEF, left ventricular ejection fraction; CK-MB, creatine kinase-MB

| **Supplementary Table 3.**  Comparisons of baseline characteristics according to acute suicidal ideation in patients with acute coronary syndrome | | | | | |
| --- | --- | --- | --- | --- | --- |
|  |  | No suicidal ideation (N = 774) | Suicidal ideation (N = 195) | Statistical coefficient^a^ | P-value |
| Serum BDNF, mean (SD) ng/mL |  | 18.0 (7.0) | 17.0 (6.9) | t = 1.845 | P = 0.065 |
| BDNF Val66Met polymorphism, N(%) Met/Met |  | 176 (22.7) | 53 (27.2) | χ^2^ = 5.864 | P = 0.053 |
| **Socio-demographic characteristics** |  |  |  |  |  |
| Age, mean (SD) years |  | 58.0 (11.3) | 58.9 (10.6) | t = -1.006 | P = 0.315 |
| Sex, N (%) female |  | 201 (26.0) | 68 (34.9) | χ^2^ = 6.156 | P = 0.013 |
| Education, mean (SD) years |  | 10.0 (4.7) | 9.1 (4.4) | t = 2.523 | P = 0.012 |
| Marital status, N (%) unmarried |  | 106 (13.7) | 35 (17.9) | χ^2^ = 2.267 | P = 0.132 |
| Living alone, N (%) |  | 71 (9.2) | 21 (10.8) | χ^2^ = 0.462 | P = 0.497 |
| Housing, N (%) rented |  | 109 (14.1) | 41 (21.0) | χ^2^ = 5.739 | P = 0.017 |
| Currently unemployed, N (%) |  | 279 (36.0) | 89 (45.6) | χ^2^ = 6.087 | P = 0.014 |
| **Laboratory assessment** |  |  |  |  |  |
| Fasting glucose, mean (SD )mg/dL |  | 134.5 (41.6) | 139.4 (52.1) | t = -1.234 | P = 0.159 |
| Total cholesterol, mean (SD) mg/dL |  | 185.8 (39.0) | 183.6 (41.4) | t = 0.695 | P = 0.487 |
| BUN, mean (SD) mg/dL |  | 15.5 (10.5) | 14.4 (5.3) | t = 1.407 | P = 0.160 |
| Creatinine, mean (SD) mg/dL |  | 0.89 (0.28) | 0.87 (0.30) | t = 0.588 | P = 0.556 |
| **Depression characteristics** |  |  |  |  |  |
| Previous depression, N (%) |  | 19 (2.5) | 15 (7.7) | χ^2^ = 12.620 | P < 0.001 |
| Family history of depression, N (%) |  | 15 (1.9) | 8 (4.1) | χ^2^ = 3.149 | P = 0.076 |
| BDI, mean (SD) score |  | 7.7 (6.4) | 19.4 (9.8) | t = -15.830 | P < 0.001 |
| **Cardiac risk factors, N (%)** |  |  |  |  |  |
| Previous ACS |  | 30 (3.9) | 9 (4.6) | χ^2^ = 0.220 | P = 0.639 |
| Family history of ACS |  | 24 (3.1) | 7 (3.6) | χ^2^ = 0.120 | P = 0.729 |
| Hypertension |  | 360 (46.5) | 98 (50.3) | χ^2^ = 0.876 | P = 0.349 |
| Diabetes |  | 144 (18.6) | 47 (24.1) | χ^2^ = 2.975 | P = 0.085 |
| Hypercholesterolemia |  | 384 (49.6) | 102 (52.3) | χ^2^ = 0.453 | P = 0.501 |
| Obesity |  | 341 (44.1) | 74 (37.9) | χ^2^ = 2.373 | P = 0.123 |
| Current smoker |  | 297 (38.4) | 69 (35.4) | χ^2^ = 0.591 | P = 0.442 |
| **Current cardiac status** |  |  |  |  |  |
| Killip class >1, N (%) |  | 132 (17.1) | 36 (18.5) | χ^2^ = 0.215 | P = 0.643 |
| LVEF, mean (SD) |  | 61.2 (11.4) | 61.1 (10.8) | t = 0.111 | P = 0.911 |
| Troponin I, mean (SD) mg/dL |  | 9.5 (15.0) | 11.5 (14.7) | t = -1.685 | P = 0.092 |
| CK-MB, mean (SD) mg/dL |  | 16.8 (38.2) | 19.7 (33.3) | t = -0.969 | P = 0.333 |

^a^Independent two-sample t-test or χ^2^ test, as appropriate. BDNF, brain-derived neurotrophic factor; BUN, blood urea nitrogen; BDI, Beck Depression Inventory; ACS, acute coronary syndrome; LVEF, left ventricular ejection fraction; CK-MB, creatine kinase-MB

**Supplementary Methods**

**Eligibility criteria for the DEPACS participants – add as Supplementary Methods**

For the DEPACS study entry, inclusion criteria were as follows: i) aged 18~85 years; ii) confirmed ACS by investigation (the presence of ST-segment elevation MI was determined by >30 min of continuous chest pain, a new ST-segment elevation ≥2 mm on at least two contiguous electrocardiographic leads, and creatine kinase-MB more than three times normal; the presence of non-ST-segment elevation MI was diagnosed by chest pain and a positive cardiac biochemical marker without new ST-segment elevation; and the presence of unstable angina was determined by chest pain within the preceding 72 h with or without ST-T wave changes or positive cardiac biochemical markers); iii) ability to complete study questionnaires; iv) ability to understand the study objectives and sign informed consent. Exclusion criteria were: i) occurrence of ACS while hospitalized for another reason; ii) ACS developing less than 3 months after a coronary artery bypass graft procedure; iii) uncontrolled hypertension (systolic blood pressure (BP) >180mmHg or diastolic BP >100mmHg), the same criteria were used in the SADHART trial ^1^; iv) resting heart rate <40/min; v) severe physical illnesses threatening life or interfering with the recovery from ACS; vi) persistent clinically significant laboratory abnormalities in complete blood cell counts, thyroid tests, renal function tests, and liver function tests.

**Polymerase chain reaction protocol for *BDNF* genotyping**

Polymerase chain reaction (PCR) and PCR-based restriction fragment length polymorphism assays were conducted. The forward and reverse primers had the sequences 5’-ACTCTGGAGAGCGTGAATGG-3’ and 5’‑ACTACTGAGCATCACCCTGGA-3’, respectively. The amplification conditions were pre-denaturation at 95°C for 5 min followed by 40 cycles of denaturation at 95°C for 30 s, 62°C for 30 s, and 72°C for 30 s, with post-elongation at 72°C for 5 min and a final maintenance step at 4°C. The PCR products were digested at 37°C with the corresponding restriction enzyme (*Eco*72I) and separated by gel electrophoresis to identify the 196G (Val: 99- and 72-bp fragments) and 196A (Met: 171-bp fragment) alleles.

**References**

1 Glassman, A. H. *et al.* Sertraline treatment of major depression in patients with acute MI or unstable angina. *JAMA* **288**, 701-709, doi:10.1001/jama.288.6.701 (2002).
